# Dynamics of following and leading: association of movement synchrony and depression severity

**DOI:** 10.3389/fpsyt.2024.1459082

**Published:** 2024-09-17

**Authors:** Simone Jennissen, Anna Sandmeir, Desiree Schoenherr, Uwe Altmann, Christoph Nikendei, Henning Schauenburg, Hans-Christoph Friederich, Ulrike Dinger

**Affiliations:** ^1^ Institute for Medical Psychology, Heidelberg University, Heidelberg, Germany; ^2^ Department of Psychosomatic Medicine and Psychotherapy, LVR Hospital Düsseldorf, Medical Faculty, Heinrich-Heine University, Düsseldorf, Germany; ^3^ Department of General Internal Medicine and Psychosomatics, Heidelberg University Hospital, Heidelberg, Germany; ^4^ Institute for Psychosocial Medicine, Psychotherapy, and Psycho-Oncology, Jena University, Jena, Germany; ^5^ Department of Psychology, Medical School Berlin (MSB) GmbH, Berlin, Germany; ^6^ Clinical Institute for Psychosomatic Medicine and Psychotherapy, Medical Faculty and University Hospital Düsseldorf, Heinrich Heine University Düsseldorf, Düsseldorf, Germany

**Keywords:** movement synchrony, nonverbal synchrony, motion energy analysis, time series analysis, depression, leading

## Abstract

**Objective:**

Depression negatively affects interpersonal functioning and influences nonverbal behavior. Interpersonal theories of depression suggest that depressed individuals engage in behaviors that initially provoke others’ support and reassurance, but eventually lead to rejection that may also be expressed nonverbally.

**Methods:**

This study investigated movement synchrony as a nonverbal indicator of support and rejection and its association with depression severity in a sample of depressed and healthy individuals. Semi-standardized diagnostic interview segments with *N* = 114 dyads were video recorded. Body movement was analyzed using Motion Energy Analysis, synchrony intervals were identified by computing windowed cross-lagged correlation and a peak-picking-algorithm. Depression severity was assessed via both self-rating (BDI-II) and clinician rating (HAMD).

**Results:**

Both self-rated and clinician-rated depression severity were negatively correlated with patient-led, but not clinician-led movement synchrony measures. The more depressed patients were, the less they initiated movement synchrony with their clinicians. These correlations remained significant after controlling for gender, age, gross body movement, and psychopharmacological medication.

**Conclusion:**

Findings suggest that depression may negatively affect patients’ active initiative in interaction situations. Automatized methods as used in this study can add valuable information in the diagnosis of depression and the assessment of associated social impairments.

## 1 Introduction

Depression is a complex disorder that impairs the afflicted individuals in many ways. In addition to affective, cognitive and psycho-vegetative symptoms, depression also extends to the social environment, often entails impairments in social functioning, and compromises relationships (1–3). Interpersonal difficulties are both a consequence and an antecedent of depression, offering a potential explanation for the self-perpetuating nature of depression (4, 5). It has been repeatedly shown that depression affects nonverbal interaction behavior (6–8). In dyadic interaction situations, depressed individuals tend to express behaviors that appear distanced, withdrawn, and uninvolved (9, 10). Depression is further associated with psychomotor slowing (11), and depression severity was found to be negatively correlated with gross body movement in clinical interviews (12).

### 1.1 Interpersonal theories of depression

Interpersonal theories of depression suggest that depressed individuals engage in behaviors that initially provoke others’ support and reassurance, but eventually lead to rejection (4, 5, 13). More specifically, due to the depressive core feeling that there’s something wrong with oneself, depressed individuals are assumed to express their helplessness and withdraw from interactions at first, which shifts the responsibility for the interaction towards the other person (4). Furthermore, they often seek reassurance that others truly care about them, while at the same time engaging in negative feedback seeking to “verify” their negative self-concept (5, 14). While others may initially provide reassurance, depressed individuals are likely to doubt their sincerity and demand further comfort, which at some point will result in frustration and anger in the other person (13). Furthermore, the inconsistency of depressed individuals to seek both reassurance and negative feedback is likely to leave others irritated as well (5). As the other person’s anger aggravates, the initially supportive environment might turn hostile and rejecting, thereby confirming the depressed individual’s negative assumptions about themselves (5).

Following from interpersonal theories, depressed individuals demand both support and negative affirmation from others. While this may elicit favorable responses by others initially, those are likely to turn into rejection and withdrawal once the other person’s anger about the continuous and inconsistent demands is exacerbated. Apart from verbal expressions of negative affect towards the depressed individual, rejection and withdrawal are likely also displayed nonverbally (15). As nonverbal communication often occurs in a fast and unconscious manner, the aforementioned interpersonal dynamics of engagement, rejection and/or withdrawal between depressed and non-depressed individuals may be expressed earlier on the level of dyadic nonverbal behavior.

### 1.2 Nonverbal synchronization

Nonverbal synchronization phenomena are a frequent occurrence in dyadic nonverbal behavior. They include various forms of temporal coordination of nonverbal behaviors between two interacting individuals (16). Nonverbal synchronization is often understood as an embodied manifestation of relationship quality (17) and an expression of interpersonal closeness (18, 19). Nonverbal synchronization phenomena like synchronous body movements or attunement of prosodic features are a mostly unintentional aspect of nonverbal human communication and have previously been described as “social glue” (20).

In recent years, the research on nonverbal synchronization phenomena has made great progress. One method to capture movement signals on a micro-level is Motion Energy Analysis (MEA; 21–25). MEA is a computer-based, objective measure of body movement, registering frame-to-frame color pixel changes of videos as several data points per second. Movement synchrony (MS) intervals are identified via correlative time series analysis methods. A MS interval is a continuous time interval with two individuals moving simultaneously or with a slight time-delay (21, 24). Several synchrony indicators can be computed (26), such as total MS, which is the amount of time a dyad spends in synchrony. This can be split into MS led by individual A (respectively followed by individual B) and MS led by individual B (respectively followed by individual A). Splitting MS into leading and following allows insight into the interactional dynamics of a dyad. When individual B follows the movement initiated by individual A, this can be interpreted as a nonverbal attempt to relate.

Regarding MS and psychopathology, it has been found that during diagnostic interviews, depressed individuals showed less MS than healthy control participants (27). During psychotherapy sessions, there was less MS in dyads with patients suffering from depressive disorders versus dyads with patients suffering from anxiety disorders (28), and early MS was negatively correlated with depression severity at the end of therapy (27). During psychotherapy with depressed patients, MS between patients and therapists increased over the course of therapy (28). Several studies highlight the relevance of differentiating leading and following regarding MS. At the beginning of therapy, patient-led MS was more strongly associated with therapy process variables such as therapeutic bond, while at the end of therapy, therapist-led MS was more strongly associated with positive treatment outcome (29). These findings suggest that at the beginning, it is beneficial that the therapist establishes a therapeutic relationship through attuning to patients by following their movements. During the termination phase, the phenomenon of patient following could reflect that the therapist functions as a role model for the patient. Similarly, studies found that higher patient-led MS in session three predicted reduced drop-out rates in a sample of patients with social anxiety disorder (25), while higher patient-led MS in session eight was associated with lower therapeutic alliance and higher symptom severity (30).

To summarize, depression seems to be associated with less MS overall, while findings regarding the dynamics of leading and following are less clear. Worded differently, it is yet to be determined whether the reduced MS between depressed patients and their clinicians stems from a diminished ability of depressed patients to initiate nonverbal synchronization with others (i.e. reduced patient-led synchrony: clinicians don’t follow their patients’ movements) or whether it is due to their own reduced reaction to clinicians’ movements (i.e. reduced clinician-led synchrony).

Taking interpersonal theories of depression into account, both reasons for reduced overall MS seem possible. On the one hand, depressed patients may demand affirmation and support on the level of nonverbal behavior, which could be met by withdrawal of clinicians (i.e. less patient-led MS; 4, 5). On the other hand, clinicians’ negative reaction towards depressed patients may result in less nonverbal engagement and depressed patients’ tendencies towards withdrawal and lethargy could prevent them from reacting to clinicians’ movements (i.e. less clinician-led MS; 4, 5). Thus, investigating the dynamics of leading and following has the potential to provide more general insight into how nonverbal dyadic behavior is altered by depression.

### 1.3 The present study

Several methodological issues regarding the investigation of MS in depression have been highlighted (27) and are being addressed in the present study. A first methodological challenge is the investigated situation. Naturalistic interaction situations like psychotherapy sessions (e.g. 31) ensure high ecological validity at the expense of standardization. As an alternative, a standardized clinical interview situation like the Structured Clinical Interview for DSM (SCID, 32) would ensure comparability of the investigated situation between patients at the expense of ecological validity, since the standardization of questions for all patients differs largely from a naturalistic psychotherapy setting (e.g. 27). In a standardized interview, the interaction is not natural, interviewers are typically preoccupied with reading questions or marking responses and the use of a manual can result in movement bias due to frequent page turning. The present study aims to balance the need for natural interactions between clinician and patient with a sufficient level of standardization by using a semi-standardized interview section to evaluate MS. In a semi-standardized interview, several questions and prompts are predefined, while at the same time there is room for additional spontaneous questions from the therapist, thereby mimicking a more natural treatment setting.

A second crucial limitation of previous studies on MS and depression is the lacking control of psychopharmacological medication. Anti-depressant medication, and especially selective serotonin reuptake inhibitors (SSRIs) are known to result in behavioral activation of patients (33). This may affect patients’ gross body movement as well as their inclination to move in synchrony with their therapist. Thus, when investigating the effects of depression on MS, it is important to control for potentially counteracting effects of antidepressant medication. To the knowledge of the authors, the present study is the first to close this gap by assessing and statistically controlling for medication.

Lastly, the present study will control for gender, since previous studies suggest that same-gender vs. mixed-gender dyads can affect synchrony such that female dyads demonstrate higher levels of synchrony than male or mixed-gender dyads (34, 35). By statistically controlling for gender, the effect of depression on MS can be assessed independent from gender differences.

Previous studies on depression and MS have focused mainly on the total amount of MS (27, 28), while less is known about the leading/following dynamic and the time-lag. By investigating the leading/following dynamics, the present study aims to further elucidate dyadic nonverbal behavior associated with depression, and to contribute to explaining interpersonal difficulties associated with depression. Considering interpersonal theories of depression and the findings reported above, we hypothesize that higher depression severity is related to less total MS, even when controlling for gender, gross body movement, and psychopharmacological medication and investigate whether this effect is due to less patient-led or less clinician-led MS. Summarizing the effects of patient-vs.-therapist lead in one variable, the leading varible of MS will be investigated. The leading variable represents the difference between patient-led and clinician led movement synchrony, with a positive value indicating that the patient has initiated more synchrony intervals than the clinician. Additionally, we will examine the time-lag, i.e. the amount of time that passes between one individual initiating a movement and the other individual following that movement and thereby creating MS. Again, the time-lag can be split into the mean time lag when patients follow clinicians (clinician-led) and the mean time lag when clinicans follow patients (patient-led). Furthermore, an exploratory analysis of associations of these MS measures with interpersonal variables (interpersonal problems, dependency, and self-criticism), as well as their respective contribution to explaining variance in MS over and above depression severity will be performed. There are no specific hypotheses regarding the exploratory variables.

## 2 Methods

The ethics committee of the Heidelberg University Hospital approved all study procedures.

### 2.1 Participants and procedure

The analyses in this cross-sectional study are based on a sample comprising a clinical and a non-clinical subsample recruited September 2018 to February 2020. The clinical sub-sample (*N* = 88) were patients recruited for the study at their admission to treatment in a psychosomatic inpatient (*n* = 68) and outpatient (*n* = 20) facility of a university hospital for psychosomatic medicine in Germany. Inclusion criteria for the clinical sub-sample were a main diagnosis of a depressive disorder (major depressive disorder, minor depressive disorder, dysthymia) according to the DSM-IV, absence of psychotic disorders, fluency in German and age > 18 years. At admission, patients were asked for their written informed consent to allow their data to be analyzed. The video recording of the diagnostic interview used for the current study was part of standard diagnostic procedures at the beginning of treatment. Patients were diagnosed according to the Structured Clinical Interview for Axis I DSM–IV Disorders (SCID-I; 32). The semi-standardized Level of Personality Functioning Interview (LPFS; 36) was used to assess personality functioning according to the alternative DSM–5 model of personality disorder classification (37). Then followed the Hamilton Depression Scale interview (HAMD; 38) and the completion of a battery of questionnaires including the ones used for analyses in the current study.

The non-clinical sub-sample (*N* = 26) was recruited via flyers posted in various public institutions and online on social media. Inclusion criteria for the non-clinical sub-sample were absence of a psychological disorder according to the DSM-IV, fluency in German, age > 18 years, and no ongoing psychotherapeutic or psychiatric treatment. Respondents to the advertisements underwent telephone screening which included the SCID-I interview to ensure that they met inclusion criteria. If respondents met inclusion criteria, they were invited to the clinic for a personal interview. The HAMD and the first eight introductory questions of the Level of Personality Functioning Interview (36) were conducted, and study questionnaires were completed. At full completion of the interview and questionnaires, the non-clinical participants received a financial compensation of 20 €. A ratio of clinical versus healthy study participants of 4:1 had been determined in the study protocol. Analyses are conducted on the whole sample unless stated otherwise.

### 2.2 Measures

#### 2.2.1 Hamilton Depression Scale

The Hamilton Depression Scale is a clinician administered interview to assess depression severity (39). In the current study the 17-item version was used. The HAMD is internationally considered the gold standard for measuring depression. Interviews were rated by a trained clinician using a rating grid. Depending on the item, a 3- to 5-point Likert scale is used ranging from 0 (symptom not present) to 2 or 4 (symptom present most of the time and/or with very high intensity; 38). After appropriate training the inter-rater reliability is good (e.g. 40). In the current sample, Cronbach’s alpha of.862 indicated high internal consistency.

#### 2.2.2 Beck Depression Inventory-II

The revised BDI-II is a widely used 21-item self-rated measure of depression severity (41). Patients rate 21 symptoms of depression regarding their severity by choosing one of four statements, corresponding to a 4-point Likert scale, from 0 = “absent or mild” to 3 = “severe”. The German version demonstrates high reliability and is strongly correlated with related measures of depression (42). In the current sample, the BDI-II demonstrated excellent reliability with a Cronbach’s alpha of.946.

#### 2.2.3 Inventory of interpersonal problems

The short version of the Inventory of interpersonal problems (IIP-32) is a self-report measure of interpersonal difficulties and distress comprising 32 items (43). It measures interpersonal styles across different situations and supplies a global measure for interpersonal difficulties. The German 32-item version of the IIP has shown satisfactory to good psychometric properties (44). In the current sample, Cronbach’s alpha of .871 indicated high internal consistency.

#### 2.2.4 Theoretical Depressive Experiences Questionnaire

The Theoretical Depressive Experiences Questionnaire – 12 Item Version (TDEQ-12) is a self-report measure assessing depressive experiences related to dependency and self-criticism, with each item rated on a 7-point Likert scale from 1 = “strongly disagree” to 7 = “strongly agree (45, 46). The Dependency scale assesses experiences associated with depression such as loneliness, helplessness and fear of rejection. The Self-Criticism scale captures facets of depression such as worthlessness, feelings inadequacy and guilt. The psychometric properties of the German version of the TDEQ-12 are satisfactory and comparable to the original 64 item version (47). In the current sample, internal consistency was acceptable to high for both the dependency subscale (Cronbach’s alpha = .846) and the self-criticism subscale (Cronbach’s alpha = .796).

#### 2.2.5 Psychopharmacological medication

Psychopharmacological medication, including information on the name of the drug and dosage, was assessed via self-report at the beginning of treatment. Since patient self-reports on exact drug names and dosage are prone to bias, for the purpose of the current investigation, this information was coded as a categorical variable with the two categories “receiving any psychopharmacological medication” or “not receiving any psychopharmacological medication”.

#### 2.2.6 Video recordings

The video recordings of the first eight introductory questions of the level of personality functioning interview (36) were used to analyze MS. During this part, the clinician asks the patient general questions about themselves, for example “How would you describe yourself as a person?” or “Who are the most important people in your life?”. This interview segment was chosen since in comparison to other parts of the diagnostic interview, the open questions allow for a less restricted and more naturalistic interaction. To avoid video artefacts, diagnosticians had been instructed not to take any notes and not to flip any pages in their interview folder. Diagnostician and patient were seated across from each other at a small table. The camera recorded both individuals from the side at an angle of approximately 90°. The interview segments were recorded with a digital camera (Panasonic HC-V180) with a resolution of 1280x720 pixels and a frame rate of 25/sec. The diagnostic interviews were conducted by eight female clinical psychology graduate students (*n*=7 B.Sc.; *n*=1 M.Sc.; mean age in years: *M* = 25.50, *SD* = 1.77) who had received previous training in conducting the diagnostic interview and were blind to research hypotheses. In order to keep interview conditions as comparable as possible and since gender has been found to influence nonverbal synchrony (34), interviews conducted by a single male clinician were excluded from analyses.

### 2.3 Motion Energy Analysis and movement synchrony identification

#### 2.3.1 Measurement of body movements via Motion Energy Analysis

Body movements of clinicians and patients^1^ were assessed automatically using the published MATLAB^©^ script for MEA (GitHub: https://github.com/10101-00001/MEA; 21, 48, 49). Body motions were coded separately for patients and clinicians. In this procedure, movement is operationalized as the number of grey scale pixels changing from one video frame to the next. We followed the processing steps as described by Altmann and Schoenherr (49) and in previous studies (25, 26). To filter video noise (e.g. subtle light changes), only pixel changes above a threshold of 3 from frame *t* to *t*+1 were being counted as movement. This cut-off value resulted from computing MEA of background pixels in which no movement takes place in 10 video sequences and then determining the 99% quantile of intensity change. All settings used in the processing of movement energy time series have been validated in previous work (24). Participants’ gross body movement was operationalized as the percentage of time of the respective interview segment they spent moving, based on the time series resulting from MEA. Resulting from the separate assessment of patient and clinician body movements, there were two person time series for patients and clinicians.

#### 2.3.2 Identification and quantification of movement synchrony

**Figure 1:**
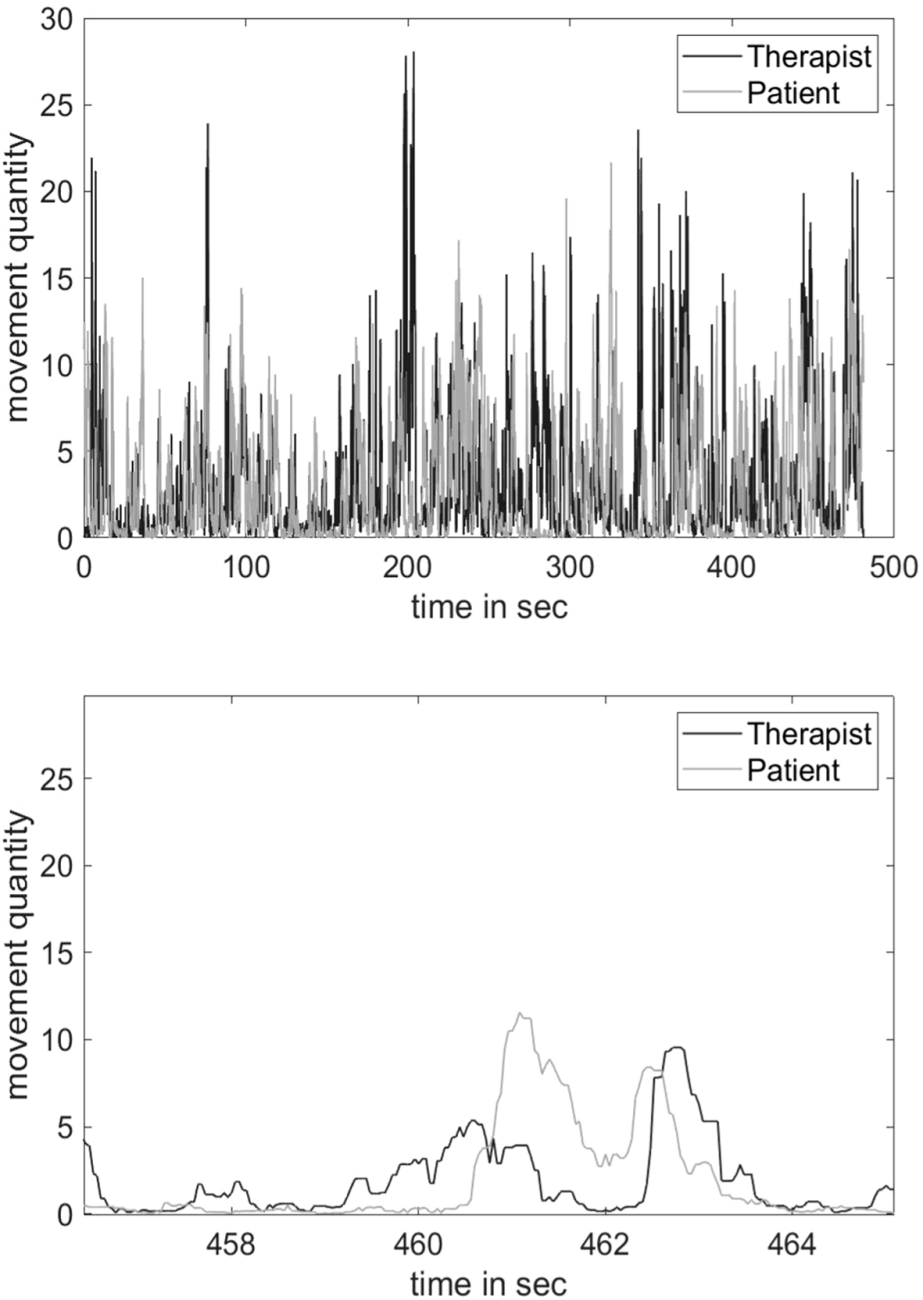
Time series of movement signals of a dyad with a total movement synchrony of MS = .55. *Note*. The upper figure shows the time series of the entire interview segment, the lower figure shows an enlarged partial segment.

On the basis on the resulting time series (for an example, see **Figure 1**), MS was computed using another MATLAB^©^ script, implementing windowed cross-lagged correlations (WCLC) of the patient and clinician time series in each video. Synchrony intervals then were identified applying a peak-picking algorithm (GitHub: https://github.com/10101-00001/sync_ident; 21, 48, 49). The algorithm correlates a window of patients’ time series with clinicians’ time series. Movements are considered synchronous up to a latency of five seconds, which is the maximum time lag set in the present WCLC analysis. In order to control for spurious correlations between the movement time series, a cut-off for *R* ^2^ values was set to.25. The five second time lag and *R* ^2^ cutoff value were empirically identified as the most suitable, as they resulted in the least amount of false positives when identifying synchrony intervals (24). After applying the algorithm, there are two resulting variables: patient-led and clinician-led MS. These can be summarized into total MS. The MS measures used in the present study are total MS, patient-led MS, clinician-led MS, leading, mean time-lag between synchrony intervals, patient-led mean time-lag, and clinician-led mean time-lag.

MS represents the portion of an interaction sequence spent in synchrony, so that 40% of an interaction sequence spent in synchrony would result in a value of .40. The leading variable is calculated by subtracting clinician-led movement synchrony from patient-led movement synchrony, with a positive value indicating that the patient has initiated more synchrony intervals than the clinician. Mean time-lag describes how quickly the two individuals react to each other with regards to synchrony. It is the mean time-lag across all synchrony intervals between one individual initiating movement and the other individual joining in, resulting in synchronous movement. Again, it can be split into patient-led mean time-lag and clinician-led mean time-lag^2^.

### 2.4 Data analysis

To address our research hypotheses, we conducted two-sided Pearson correlations between total MS, patient-led MS, and clinician-led MS and the depression severity measures (BDI-II, HAMD). We then repeated these correlations while controlling for participants’ gross body movement, gender, age, and psychopharmacological medication. Gross body movement was added as a control variable since it has been shown to correlate negatively with depression severity (12). Patient gender was added as a control variable since previous studies suggest that same-gender vs. mixed-gender dyads can influence synchrony (34, 35). Age was added as a control variable since in preliminary data analyses the non-clinical sub-sample emerged as significantly younger than the clinical sub-sample. Psychopharmacological medication was added as a control variable due to its potential psychomotor side effects.

To gain a more comprehensive picture of movement synchrony dynamics, we further calculated correlations between depression severity and the leading variable as well as time-lag. Additionally, we conducted exploratory two-sided Pearson correlations between our MS measures and dependency (TDEQ-12), self-criticism (TDEQ-12), and interpersonal problems (IIP-32). We then proceeded with a block-wise multiple regression analysis to examine the relative predictive influence of depression severity and interpersonal variables on MS. Based on the results from the bivariate correlations, we chose patient-led MS as our criterion variable and patients’ age, gender, medication, gross body movement, depression severity (HAMD) and TDEQ-12 dependency as our predictors. We chose HAMD as our predictor since it is considered the gold standard for assessment of depression severity.

## 3 Results

Of 205 patients that were admitted and had undergone the routine diagnostic session during the recruitment period for the study, 88 were included into the study for the clinical sub-sample. Forty-eight were excluded since they did not meet the inclusion criterion of a depressive disorder; 24 did not want to participate in the study; 23 had their diagnostic interview conducted by a male clinician and were thus excluded to ensure standardization of the diagnostic situation; 11 had their diagnostic interview conducted by a clinician involved in the present study and were excluded to ensure blinding to hypotheses; and for 11 participants, no suitable video for analysis was available, due to technical difficulties during video recording. Regarding the non-clinical sub-sample, of the 27 participants completing the in-person interview, one had to be excluded from the study due to technical errors during video recording.

### 3.1 Sample characteristics

For study participant characteristics, see **Table 1**. The non-clinical sub-sample was significantly younger (*p* <.01) than the clinical sub-sample. The clinical sub-sample scored significantly higher on both measures of depression severity (BDI-II: *M* = 25.97, *SD* = 10.52; HAMD: *M* = 17.90, *SD* = 6.21) than the non-clinical sub-sample (BDI-II: *M* = 2.38, *SD* = 1.60; HAMD: *M* = 2.31, *SD* = 1.70; *p* <.001). For means and standard deviations of the study variables, see **Table 2**. Analyzed video segments (*N* = 114) were between 4:24 and 25:32 minutes long (*M* = 11:03; *SD* = 3:49). There was no significant difference regarding video segment length between the clinical and the non-clinical sub-sample (*p* = .193). Due to a change in the intake standard diagnostic procedure during the study period, 15 (13.2%) study participants did not complete the TDEQ-12.

**Table 1:** Sociodemographic characteristics of study participants.

Variables						
	Full Sample *N* = 114		Clinical Sub-Sample *N* = 88		Non-Clinical Sub-Sample *N* = 26	
Mean Age in years	34.4 (*SD* = 12.43)		36.02 (*SD* = 12.49)		28.96 (*SD* = 10.75)	
	*N*	*N* in %	*N*	*N* in %	*N*	*N* in %
Gender						
Female	77	67.5	60	68.2	17	65.4
Male	37	32.5	28	31.8	9	34.6
Marital status						
Single	47	41.6	37	42.5	10	38.5
Married/partnered	57	50.4	43	49.4	14	53.9
Divorced/widowed	9	8.0	7	8.0	2	7.7
Highest educational level						
Basic secondary school	30	26.3	26	29.5	4	15.4
Higher secondary school with university entrance qualification	30	26.3	21	23.9	9	34.6
University education	54	47.4	41	46.6	13	50.0
Depressive disorders						
Major depressive disorder			75	85.2		
Minor depressive disorder			8	9.1		
Dysthymia			16	18.2		
Other disorders						
Substance related disorders			7	7.9		
Anxiety disorders			44	50.0		
Somatoform disorders			21	23.9		
Eating disorders			15	17.0		
Psychopharmacological medication			35	40.2		
Anti-depressive medication			33	37.9		
Other psychopharmacological medication			12	13.8		

For n = 1 participant (0.9%), information on marital status was missing, for n = 1 (0.9%) participant, information on psychopharmacological medication was missing. For n = 3 (2.7%) participants, BDI-II data were missing, for n = 1 (0.9%) participant, HAMD data were missing, and for n = 4 (3.6%) participants, IIP-32 data were missing.

**Table 2:** Means, standard deviations and range of study variables for the complete sample of N = 114 participants (n = 88 clinical and n = 26 non-clinical).

Variable	M	SD	Range	N
HAMD	14.28	8.60	[0; 32]	112
BDI-II	20.54	13.60	[0; 50]	113
IIP-32_Global	1.54	.54	[.28; 2.88]	110
TDEQ-12 Dependency	3.92	1.49	[1.40; 7.00]	96^3^
TDEQ-12 Self-criticism	4.36	1.37	[1.57; 7.00]	98
Movement Synchrony	.55	.04	[.43;.63]	114
Patient-led	.28	.03	[.19;.36]	114
Clinician-led	.27	.03	[.19;.35]	114
Leading Variable	0.01	.05	[-.09;.12]	114
Mean Time-lag^1^	2.49	0.11	[2.24; 2.72]	113^2^
Patient-led	2.47	0.16	[2.05; 2.87]	114
Clinician-led	2.54	0.13	[2.11; 2.8]	114

^1^In seconds. ^2^One case was excluded with an extreme value, z > -3.29. ^3^Numbers vary slightly due to missing data.

### 3.2 Main results

**Table 3** shows the zero-order correlations between MS measures and depression severity (BDI-II, HAMD). Regarding our hypothesis, there was a significant negative correlation of total MS with both clinician-rated (*r*_HAMD_ = -.342**) and patient-rated depression severity (*r*_BDI-II_ = -.346**), which was still present after adding the control variables (*r*_HAMD_ = -.247* and *r*_BDI-II_ = -.276*, respectively). There was no significant correlation between clinician-led MS and depression severity (*r* range from *r* = -.062 to *r* = .001, *n.s.*). However, there was a significant negative correlation of patient-led MS and depression severity (*r*_HAMD_ = -.337**, *r*_BDI-II_ = -.340**), even after adding the control variables (*r*_HAMD_ = -.262**, *r*_BDI-II_ = -.278**). Additionally, a significant negative correlation was observed between depression severity and the leading variable (*r*_HAMD_ = -.194*, *r*_BDI-II_ = -.200*), which was marginally significant after adding the control variables (*r*_HAMD_ = -.175^+^, *r*_BDI-II_ = -.178^+^) There was also a significant positive correlation between HAMD and patient-led time lag (*r* = .217*), even after adding the control variables (*r* = .190*). There was no significant correlation between depression severity and clinician-led time-lag (*r* range from *r* = .050 to *r* = .134, *n.s.*).

**Table 3:** Correlations and partial correlations between depression severity, interpersonal problems, dependency, self–criticism and movement synchrony measures for the complete sample of N = 114 participants (n = 88 clinical and n = 26 non-clinical).

Zero-Order Pearson correlations							
Variable	Movement Synchrony			Leading	Mean time-lag		
	Total	Pat	Clin		Total	Pat	Clin
HAMD	–.342**	–.337**	–.062	–.194*	.202*	.217*	.054
BDI-II	–.346**	–.340**	–.056	–.200*	.191*	.158	.134
IIP-32 Global	–.128	–.212*	.093	–.199*	.172	.189*	.127
TDEQ-12 Dependency	–.246*	–.330**	.048	–.255*	0.168	.065	.152
TDEQ-12 Self-criticism	–0.170	–.183	–.005	–.125	0.134	.025	.170
Partial correlations with statistical control of age, gender, psychopharmacological medication, gross body movement							
HAMD	–.247*	–.262**	.001	–.175^+^	.177^+^	.190*	.050
BDI-II	–.276**	–.278**	–.011	–.178^+^	.169^+^	.125	.132
IIP-32 Global	–.085	–.176^+^	.117	–.183^+^	.142	.148	.116
TDEQ-12 Dependency	–.126	–.258*	.140	–.249*	.152	.040	.154
TDEQ-12 Self-criticism	–.097	–.115	.027	–.092	.092	–.034	.169

HAMD, Hamilton Depression Rating Scale (clinician rating); BDI-II, Beck Depression Inventory-II; IIP-32 Global, Short Version of the Inventory of Interpersonal Problems; TDEQ-12, Theoretical Depressive Experiences Questionnaire-12 Item Version; pat, patient-led; clin, clinician-led; leading = (movement synchrony patient-led – movement synchrony clinician-led); Negative leading values mean that X is more often the leader when synchronizing than Y; *p <.05, two-tailed. **p <.01, two-tailed. ^+^p <.10, two-tailed.

### 3.3 Exploratory analyses

Regarding our exploratory research questions, significant negative correlations were found only between dependency and patient-led MS (*r* = -.258*) as well as the leading variable after adding the control variables (*r* = -.249*) **Table 3**). We additionally conducted separate analyses for the clinical and non-clinical sub-sample, for results see **Supplementary Materials**. The overall patterns of results for the clinical sub-sample are paralleling the results for the full sample. Due to the small size of the non-clinical sub-sample and the small variance on the clinical measures, the somewhat diverging findings here should be interpreted with caution.

To address our exploratory research question whether dependency explains variance in MS over and above depression severity, we conducted block-wise regression analyses (**Table 4**). After adding all control variables in the first model, clinician rated depression severity (HAMD) was entered as a predictor variable in our second model, and dependency as a predictor variable in our third model. Of the control variables, only gross body movement was a significant predictor of MS. Including depression severity in the second model significantly improved model fit, with gross body movement and depression severity as significant predictors. The inclusion of dependency in model three did not significantly improve model fit, and dependency was no significant predictor of MS when simultaneously accounting for depression severity.

**Table 4:** Regressions of associations between synchrony and average levels of depression severity and dependency for the complete sample of N = 114 participants (n = 88 clinical and n = 26 non-clinical).

Criterion Variable: Movement Synchrony Patient-lead									
	Model 1			Model 2			Model 3		
	*β*	*t*	*p*	*β*	*t*	*p*	*β*	*t*	*p*
Control Variables									
Age	–0.131	–1.295	.199	–0.055	–.528	.599	–0.096	–.910	.365
Gender	–0.009	–.092	.927	0.001	.010	.992	0.030	.308	.759
Patient Gross Body Movement	0.324**	3.233	.002	0.275**	2.754	.007	0.264**	2.661	.009
Psychopharmacological Medication	–0.109	–1.072	.287	–0.010	–.096	.924	0.028	.255	.799
Predictor Variables									
HAMD				–0.274*	–2.419	.018	–0.182	–1.435	.155
TDEQ-12 Dependency							–0.190	–1.589	.116
*F*	3.675**			4.270**			4.041**		
*F* Change	3.675**			5.851*			2.526		
Adjusted *R* ^2^	0.103**			0.150*			0.164		

N = 94. Model 2 is compared against Model 1, Model 3 is compared against Model 2. β = Standardized Beta-coefficients; HAMD, Hamilton Depression Rating Scale (clinician rating); TDEQ-12, Theoretical Depressive Experiences Questionnaire-12 Item Version; inclusion criterion for predictor variables to the regression model = p <.05, exclusion criterion for predictor variables = p >.10; *p <.05, two-tailed. **p <.01, two-tailed. ***p <.001, two-tailed.

## 4 Discussion

This study aimed to gain a comprehensive picture of the effects of depression severity on synchrony in dyadic nonverbal behavior. It investigated associations between depression severity and measures of MS, focusing on the dynamics of leading and following. Explorative analyses further investigated associations of MS with the interpersonal dimensions of dependency, self-criticism, and interpersonal problems. The analyzed interaction situation was a semi-standardized clinical interview segment conducted with depressed and healthy subjects, covering the entire range of depression severity.

As hypothesized, results showed that higher clinician rated (HAMD) and patient rated (BDI-II) depression severity was associated moderately with fewer synchrony intervals in total. While clinician-led MS was not related to patients’ depression severity, depression severity and MS were associated due to a lower number of synchrony intervals led by patients’ movement. Spoken differently, clinicians did not “follow” depressed patients’ movements with movements of their own. These results were stable even after controlling for gender and medication. This suggests that independent from whether dyads were of mixed (male patient, female diagnostician) or same gender (both females), there were significant associations between depression severity and patient-led MS. The previously reported higher amount of MS between female dyads did not explain the association. The effect of medication may be interpreted similarly. Whether patients take medication or not had no effect on the association between MS and depression, albeit medication may positively affect the overall amount of body movements displayed by patients.

In line with these findings, both measures of depression severity were also negatively correlated with the leading variable. The leading variable indicates to which extent patient-led MS exceeds clinician-led MS. Additionally, patient-led time lag was significantly negatively correlated with depression severity. Our results on total MS and depression severity are in line with the findings of Altmann and colleagues (27), who found a negative correlation (*r* = –.57, *p* <.001, *N* = 30) between total MS and depression severity in a sample of depressed and healthy individuals during a diagnostic interview. Notably, in our study depression severity was correlated with patient-led, but not clinician-led MS, as well as with patient-led, but not clinician-led time lag. To the knowledge of the authors, this is the first published finding regarding the time lag of MS in a sample of depressed patients.

Our findings suggest that while depression appears not to affect the patients’ ability to react to another person’s bodily movements and establish MS, it reduces the extent to which MS is being initiated by the patient. This could be interpreted as a lack of active initiative in an interaction situation, which is in line with the withdrawn and distant nonverbal behavior other studies have found to be associated with depression (9, 10). It should be pointed out however that the correlations between MS measures and depression severity in the present study are statistically controlled for gross body movement, hence reduced MS cannot solely be due to psychomotor retardation associated with depression.

Since patient-led MS is equivalent to “clinician-followed MS”, the negative correlation between patient-led MS and depression severity may also be interpreted as clinicians’ reduced inclination to nonverbally follow more severely depressed patients. The examination of the time-lag showed that the more severely depressed patients were, the longer it took clinicians to react to their movements when establishing synchrony. This finding echoes Tellenbach’s clinical observation that depression not only “lames its victims, but others who have to do with them” (50, p. xxi). Depression’s dampening effect on the nonverbal reactions of interaction partners could thus be seen as a nonverbal manifestation of the difficulties to establish contact and connection with depressed individuals. Connecting these results with interpersonal theories of depression, depressed individuals may elicit a negative response from their social environment more promptly on the level of mostly unconscious nonverbal interactions than on the level of explicit actions of rejection and withdrawal (4, 5). More precisely, depressed individuals’ demands for both reassurance and negative feedback to verify their critical view of themselves is likely to irritate others, which may show rapidly on the level of nonverbal dyadic synchronization (13).

This interpretation is further supported by the results of our exploratory analyses which demonstrated a significant negative correlation of dependency with patient-led MS as well as with the leading variable, even after adding the control variables. Hence, the higher individuals scored on dependency, the less the clinician reacted in synchrony to their movements. Similar to the findings on depression severity, there was no significant correlation between dependency and clinician-led MS, suggesting that dependency does not significantly influence individuals’ synchronous reaction to an interaction partner’s movements. Dependency manifests itself in submissiveness, excessive friendliness and low assertiveness (51), as well as in needy behavior and an increased need for support and reassurance. The latter can ultimately lead to rejection by the social environment (52, 53). The negative association we found between patient-led MS and dependency in our study could thus be tentatively interpreted as high dependency eliciting nonverbal rejection in clinicians. However, it is important to note that the exploratory stepwise regression analyses showed that dependency did not significantly predict patient-led MS over and above depression severity. The high correlation between depression severity (HAMD) and dependency (*r* = .56, *p* <.001) poses a challenge to the assessment of their separate versus shared variance with MS. Notably, there was no association between self-criticism and MS, although self-criticism is just as strongly correlated with depression as dependency (*r* = .66, *p* <.001). One possible explanation for this finding is that dependency describes a phenomenon that is more interpersonal in nature, while self-criticism is more intra-personal. This is in line with the conclusion of Schoenherr and colleagues (27), who suggested that MS is more strongly related to interpersonal characteristics than to individual symptomatology.

Since this study investigated pre-treatment diagnostic interviews, further research is needed to investigate whether findings can be replicated in psychotherapy settings with implications for clinical practice and therapeutic relationships between clinicians and depressed patients. Following from this study’s findings, it might be expected that depressed patients are less likely to initiate MS with their therapist and therapists may be less likely to follow depressed patients’ movements. This finding has important implications, since this interaction pattern of withdrawal of the depressed person’s interaction partner may further reinforce depression, thereby perpetuation the downward spiral of depression. Thus, if our study’s finding is replicated in a psychotherapy setting, clinicians might be instructed to counteract their automatic withdrawal reaction and focus on nonverbal engagement with their patients. However, before such conclusions can be drawn, the study needs to be confirmed in a preferably longitudinal study on outpatient treatment to assess whether patients and therapists demonstrate the same nonverbal behavior as patients and diagnosticians and preferably also conduct a longitudinal analysis to investigate whether improved MS in the beginning of treatment positively affects later symptoms of depression.

### 4.1 Limitations and strengths

Several limitations are to be considered in the present study. Since MS is influenced by both interactants of a dyad, MS might also vary between clinicians. Our data structure and study design with only eight clinicians did not allow for the reliable estimation of random effects in a Multilevel Analysis in order to investigate clinician effects (54). However, clinicians were very similar regarding sociodemographic variables, which could plausibly reduce variance between them^3^.

Another methodological challenge in this study was the high correlation (*r* = .52, *p* <.001) between the predictors (HAMD, TDEQ-12 Dependency) in the exploratory regression model. Additionally, the methods applied in this study only captures quantitative aspects of total movement in the predefined region of interest, while disregarding qualitative aspects such as speed, direction, or which body parts were moved.

The uniqueness and strengths of this study include the semi-standardized interview situation analyzed. In the classical dilemma between internal validity (i.e. high standardization of content and situation) and external validity (i.e. natural interaction), the study balances both in this semi-standardized interview employing therapy-relevant questions regarding the self and important relationships. We also included both a patient-rated and a clinician-rated instrument to measure depression severity in order to detect potential differences in their associations with MS measures. Furthermore, to the knowledge of the authors this is the first study to statistically control for psychopharmacological medication in a sample of depressed patients. However, due to reliability issues in the self-assessment of medication (exact drug names and dosage), medication could only be assessed dichotomously.

### 4.2 Conclusion

This study adds to previous research on characteristics of nonverbal dyadic interaction in different diagnostic groups (27, 28, 35, 55, 56) by investigating the associations between several MS measures and depression severity. This study highlights the importance of not only investigating global measures of MS, but considering finer-grain aspects such as leading and following. From a clinical perspective, this study suggests that applying MEA to diagnostic interviews can provide insight into embodied interaction dynamics with depressed individuals. Routine assessment of MS during diagnostic interviews could contribute to a more differentiated diagnosis and may allow treatment to be tailored to patient’s specific interpersonal difficulties.

## Data Availability

As per restrictions by the ethics committee, data are not allowed to be shared. Requests to access the datasets should be directed to Simone.Jennissen@med.uni-heidelberg.de.

## Footnotes

1 Unless otherwise stated, “patients” will refer to the full sample of patients and healthy control participants to avoid confusion regarding the dyads.

2 “Movement synchrony” is equivalent to “ratio_sync” in the MATLAB© script by Altmann, “patient led movement synchrony” is equivalent to “ratio_patlead”, “clinician led movement synchrony” to “ratio_therlead”, “mean time-lag” to “mean_TL” (GitHub: https://github.com/10101-00001/sync_ident; 21, 48).

3 Additionally, we conducted ANOVAs regarding all MS measures investigated in this study. No significant differences between diagnosticians regarding those MS measures were found.
