# Supplementary material for: Dynamics of following and leading: association of movement synchrony and depression severity
Source: Front Psychiatry. 2024 Sep 17;15:1459082. doi: 10.3389/fpsyt.2024.1459082 (PMC11442365; doi:10.3389/fpsyt.2024.1459082)
Supplement: Supplementary file 1 [file DataSheet1.zip › Supplementary Table 1.DOCX]

**Supplementary Material**

**Supplementary Table 1**

*Means, Standard Deviations and Range of Study variables in the clinical sub-sample*

| Variable | *M* | *SD* | *Range* | *N* |
| --- | --- | --- | --- | --- |
| HAMD | 17.90 | 8.60 | [3; 32] | 86 |
| BDI-II | 26.00 | 13.60 | [5; 50] | 87 |
| IIP-32_Global | 1.72 | .54 | [0.53; 2.88] | 84 |
| TDEQ-12 Dependency | 4.30 | 1.49 | [1.40; 7.00] | 71^2^ |
| TDEQ-12 Self-criticism | 4.82 | 1.37 | [1.71; 7.00] | 72^2^ |
| Movement Synchrony | .55 | .04 | [.43; .62] | 88 |
| Patient-led | .28 | .03 | [.19; .34] | 88 |
| Clinician-led | .27 | .03 | [.19; .33] | 88 |
| Leading Variable | 0.01 | .05 | [-.09; .11] | 88 |
| Mean Time-lag^1^ | 2.50 | 0.11 | [2.24; 2.72] | 88 |
| Patient-led | 2.48 | 0.16 | [2.05; 2.87] | 88 |
| Clinician-led | 2.55 | 0.13 | [2.11; 2.8] | 88 |

*Note.* ^1^In seconds. ^2^Numbers vary slightly due to missing data.
