# Supplementary material for: Dynamics of following and leading: association of movement synchrony and depression severity
Source: Front Psychiatry. 2024 Sep 17;15:1459082. doi: 10.3389/fpsyt.2024.1459082 (PMC11442365; doi:10.3389/fpsyt.2024.1459082)
Supplement: Supplementary file 1 [file DataSheet1.zip › Supplementary Table 2.DOCX]

**Supplementary Material**

**Supplementary Table 2**

*Means, Standard Deviations and Range of Study variables in the non-clinical sub-sample*

| Variable | *M* | *SD* | *Range* | *N* |
| --- | --- | --- | --- | --- |
| HAMD | 2.31 | 1.69 | [0; 6] | 26 |
| BDI-II | 2.38 | 1.60 | [0; 5] | 26 |
| IIP-32_Global | 0.94 | .39 | [0.28; 1.88] | 26 |
| TDEQ-12 Dependency | 2.86 | 1.12 | [1.40; 5.40] | 25 |
| TDEQ-12 Self-criticism | 3.08 | 1.13 | [1.57; 5.57] | 26 |
| Movement Synchrony | .57 | .04 | [.49; .63] | 26 |
| Patient-led | .29 | .03 | [.25; .36] | 26 |
| Clinician-led | .27 | .03 | [.21; .35] | 26 |
| Leading Variable | 0.02 | .05 | [-.08; .12] | 26 |
| Mean Time-lag^1^ | 2.44 | 0.13 | [2.08; 2.67] | 26 |
| Patient-led | 2.42 | 0.18 | [2.05; 2.75] | 26 |
| Clinician-led | 2.50 | 0.15 | [2.11; 2.73] | 26 |
