# Supplementary material for: Dynamics of following and leading: association of movement synchrony and depression severity
Source: Front Psychiatry. 2024 Sep 17;15:1459082. doi: 10.3389/fpsyt.2024.1459082 (PMC11442365; doi:10.3389/fpsyt.2024.1459082)
Supplement: Supplementary file 1 [file DataSheet1.zip › Supplementary Table 3.DOCX]

**Supplementary Material**

**Supplementary Table 3**

*Correlations and Partial Correlations between Depression Severity, Interpersonal Problems, Dependency, Self–criticism and Movement Synchrony Measures in the clinical sub-sample*

|  | Zero-Order Pearson correlations | | | | | | |
| --- | --- | --- | --- | --- | --- | --- | --- |
|  | Movement Synchrony | | | Leading | Mean time-lag | | |
| Variable | Total | Pat | Clin |  | Total | Pat | Clin |
| HAMD | –.298** | –.296** | –.044 | –.181^+^ | .075 | .136 | –.108 |
| BDI-II | –.334** | –.306** | –.073 | –.170 | .090 | .059 | .039 |
| IIP-32 Global | –.077 | –.164 | .112 | –.179 | .043 | .069 | –.032 |
| TDEQ-12 Dependency | –.167 | –.277* | .102 | –.258* | 0.192 | .100 | .122 |
| TDEQ-12 Self-criticism | –0.063 | –.164 | .147 | –.205^+^ | 0.117 | .048 | .068 |
|  | Partial correlations with statistical control of gender, medication, gross body movement | | | | | | |
| HAMD | –.145 | –.217^+^ | .107 | –.205^+^ | .099 | .152 | –.099 |
| BDI-II | –.294** | –.273* | –.013 | –.179 | .079 | .050 | .027 |
| IIP-32 Global | –.065 | –.158 | .143 | –.184 | .036 | .056 | –.027 |
| TDEQ-12 Dependency | –.093 | –.247* | .195 | –.272* | .184 | .094 | .113 |
| TDEQ-12 Self-criticism | –.109 | –.189 | .138 | –.203^+^ | .050 | .011 | .011 |

*Note*. *N* = 88; HAMD = Hamilton Depression Rating Scale (clinician rating); BDI-II = Beck Depression Inventory-II; IIP-32 Global = Short Version of the Inventory of Interpersonal Problems; TDEQ-12 = Theoretical Depressive Experiences Questionnaire-12 Item Version; pat = patient-led; clin = clinician-led; leading = (movement synchrony patient-led – movement synchrony clinician-led); **p* < .05, two-tailed. ***p* < .01, two-tailed. ^+^*p* < .10, two-tailed.
