# Supplementary material for: Dynamics of following and leading: association of movement synchrony and depression severity
Source: Front Psychiatry. 2024 Sep 17;15:1459082. doi: 10.3389/fpsyt.2024.1459082 (PMC11442365; doi:10.3389/fpsyt.2024.1459082)
Supplement: Supplementary file 1 [file DataSheet1.zip › Supplementary Table 4.DOCX]

**Supplementary Material**

**Supplementary Table 4**

*Correlations and Partial Correlations between Depression Severity, Interpersonal Problems, Dependency, Self–criticism and Movement Synchrony Measures in the non-clinical sub-sample*

|  | Zero-Order Pearson correlations | | | | | | |
| --- | --- | --- | --- | --- | --- | --- | --- |
|  | Movement Synchrony | | | Leading | Mean time-lag | | |
| Variable | Total | Pat | Clin |  | Total | Pat | Clin |
| HAMD | –.363^+^ | –.321 | –.118 | –.121 | .025 | .123 | –.104 |
| BDI-II | .185 | –.052 | .280 | –.207 | –.148 | –.067 | –.203 |
| IIP-32 Global | .313 | .135 | .250 | –.075 | .255 | .188 | .208 |
| TDEQ-12 Dependency | –.166 | –.204 | –.018 | –.112 | –.312 | –.420* | –.010 |
| TDEQ-12 Self-criticism | .011 | .264 | –.284 | .338^+^ | –0.292 | .515** | .181 |
|  | Partial correlations with statistical control of gender and gross body movement | | | | | | |
| HAMD | –.353^+^ | –.288 | –.150 | –.078 | –.196 | –.123 | –.164 |
| BDI-II | .150 | –.085 | .259 | –.212 | –.261 | –.114 | –.323 |
| IIP-32 Global | .270 | .096 | .224 | –.083 | .189 | .165 | .115 |
| TDEQ-12 Dependency | –.149 | –.262 | .072 | –.201 | –.092 | –.245 | .154 |
| TDEQ-12 Self-criticism | .038 | .272 | –.246 | .315 | –.244 | –.524* | .289 |

*Note*. *N* = 26; HAMD = Hamilton Depression Rating Scale (clinician rating); BDI-II = Beck Depression Inventory-II; IIP-32 Global = Short Version of the Inventory of Interpersonal Problems; TDEQ-12 = Theoretical Depressive Experiences Questionnaire-12 Item Version; pat = patient-led; clin = clinician-led; leading = (movement synchrony patient-led – movement synchrony clinician-led); **p* < .05, two-tailed. ***p* < .01, two-tailed. ^+^*p* < .10, two-tailed
